# Proteomic Analysis Reveals a Novel Therapeutic Strategy Using Fludarabine for Steroid-Resistant Asthma Exacerbation

**DOI:** 10.3389/fimmu.2022.805558

**Published:** 2022-02-25

**Authors:** Xiaoming Liu, Xiang Li, Ling Chen, Alan Chen-Yu Hsu, Kelly L. Asquith, Chi Liu, Karen Laurie, Ian Barr, Paul S. Foster, Ming Yang

**Affiliations:** ^1^ School of Biomedical Sciences and Pharmacy, College of Health, Medicine and Wellbeing, University of Newcastle, Callaghan, NSW, Australia; ^2^ Priority Research Centre for Health Lungs, Hunter Medical Research Institute (HMRI), University of Newcastle, New Lambton Heights, NSW, Australia; ^3^ School of Medicine and Public Health, College of Health, Medicine and Wellbeing, University of Newcastle, Callaghan, NSW, Australia; ^4^ Programme in Emerging Infectious Diseases, Duke-National University of Singapore (NUS) Medical School, Singapore, Singapore; ^5^ Department of Physiology, School of Basic Medicine Science, Central South University, Changsha, China; ^6^ WHO Collaborating Centre for Reference and Research on Influenza, The Peter Doherty Institute for Infection and Immunity, Melbourne, VIC, Australia

**Keywords:** influenza infection, asthma exacerbation, steroid-resistance, inflammation, proteomics, pathway analysis, STAT1 and fludarabine

## Abstract

Virus-induced asthma exacerbation is a health burden worldwide and lacks effective treatment. To better understand the disease pathogenesis and find novel therapeutic targets, we established a mouse model of steroid (dexamethasone (DEX)) resistant asthma exacerbation using ovalbumin (OVA) and influenza virus (FLU) infection. Using liquid chromatography-tandem mass spectrometry (LC-MC/MS), we performed a shotgun proteomics assay coupled with label-free quantification to define all dysregulated proteins in the lung proteome of asthmatic mice. Compared to control, 71, 89, and 30 proteins were found significantly upregulated by at least two-fold (p-value ≤ 0.05) in OVA-, OVA/FLU-, and OVA/FLU/DEX-treated mice, respectively. We then applied a Z-score transformed hierarchical clustering analysis and Ingenuity Pathway Analysis (IPA) to highlight the key inflammation pathways underlying the disease. Within all these upregulated proteins, 64 proteins were uniquely highly expressed in OVA/FLU mice compared to OVA mice; and 11 proteins were DEX-refractory. IPA assay revealed two of the most enriched pathways associated with these over-expressed protein clusters were those associated with MHC class I (MHC-I) antigen-presentation and interferon (IFN) signaling. Within these pathways, signal-transducer-and-activator-of-transcription-1 (STAT1) protein was identified as the most significantly changed protein contributing to the pathogenesis of exacerbation and the underlying steroid resistance based on the label-free quantification; and this was further confirmed by both Parallel Reaction Monitoring (PRM) proteomics assay and western blots. Further, the pharmacological drug Fludarabine decreased STAT1 expression, restored the responsiveness of OVA/FLU mice to DEX and markedly suppressed disease severity. Taken together, this study describes the proteomic profile underpinning molecular mechanisms of FLU-induced asthma exacerbation and identifies STAT1 as a potential therapeutic target, more importantly, we provided a novel therapeutic strategy that may be clinically translated into practice.

## Introduction

Asthma is a chronic inflammatory airway disorder, characterized by wheezing, shortness of breath, coughing, and tightness of the chest (1). Asthma exacerbation is an acute worsening of normal asthma symptoms, which significantly decreases patients’ quality-of-life and remains as a major cause of disease mortality in patients (2). The socioeconomic cost of asthma exacerbation is a significant healthcare burden globally (3).

The most common triggers of asthma exacerbation are respiratory viral infections (4). Rhinovirus (RV) and respiratory syncytial virus (RSV) are two of the most common viral pathogens causing asthma exacerbation (4). Exacerbations are characterized by the induction of an exaggerated neutrophilic inflammation on the background of an underlying type-2 immune response (known as Th2 response), regulated by cytokines secreted from innate lymphoid cells type-2 and CD4^+^ T-helper-2 lymphocytes (Th2 cells) (5). Although influenza virus (FLU)-induced asthma exacerbation occurs less frequently, it is more refractory due to a poorer response to current mainstay treatments (i.e steroid therapy) compared to RSV and RV (6). Effective clinical management of viral induced steroid-resistant asthma exacerbation remains a significant unmet need. Therefore, there is an urgent need for the development of innovative therapeutic strategies to treat asthma exacerbation, especially during those linked to FLU seasons and/or influenza pandemics.

Understanding of the underlying mechanisms of disease is key to develop therapeutic approaches. Herein, to gain insight into the molecular mechanisms operating during asthma exacerbation, we established a mouse model of FLU-induced exacerbation of asthma. This model mimics key clinical features of the steroid-resistant severe asthma phenotype. Liquid chromatography coupled with tandem mass spectrometry (LC-MS/MS) and multiple bioinformatic software platforms were then used to analyze the lung proteome during asthma exacerbation. The proteins regulating MHC class I (MHC-I) antigen-presentation and interferon (IFN) signaling pathways were highly enriched, while signal-transducer-and-activator-of-transcription-1 (STAT1) was among the most significantly changed protein within these pathways. Furthermore, we showed that pharmacological inhibition of STAT1 with Fludarabine (currently employed clinically for cancer therapy) coupled with dexamethasone (DEX) treatment significantly reduced disease severity. Overall, our study provides a novel insight into the molecular mechanisms of FLU-induced asthma exacerbation and suggests a potential new clinically adaptable therapeutic approach.

## Materials And Methods

### Mouse Asthma Models

BALB/c wild type (WT) mice were sourced from the Animal Services Unit facility and housed in a specific pathogen-free environment.

MDCK (Madin-Darby Canine Kidney) cells were used to produce the influenza virus (strain A/Puerto Rico/8/1934 H1N1) as previously described (7). Plaque assay was used for titration, which was calculated as plaque-forming units (PFU) per ml (8). For the allergic asthma model, 6-8-week-old BALB/c mice were sensitized with 50 µg ovalbumin (OVA, Sigma-Aldrich, USA) on day 0 by intraperitoneal (i.p.) injection (or Phosphate-buffered saline (PBS) for controls) and challenged by exposure to nebulized 1% OVA solution for 30 minutes/day from day 13-16. For the asthma exacerbation model, intranasal (i.n.) FLU inoculation (100 PFU/mouse) was added on day 19. Where indicated, mice were treated with Dexamethasone (DEX) (1 mg/kg, Sigma-Aldrich) in 200 µl of PBS by i.p. injection on days 20 and 22, and/or Fludarabine (Flud) (0.75mg/mouse, Selleck) in 200 µl of PBS from day 19-23. All endpoints were assessed 5 days post-infection (dpi) (day 24).

### Measurement of Lung Function and Sample Collection

Lung function (airway hyperresponsiveness, AHR) was determined using a Flexivent apparatus (FX1 system; Scireq, Montreal, Canada) to measure airway resistance in response to incremental increases of methacholine (MCh, Sigma-Aldrich) as described previously (9). Bronchoalveolar lavage fluid (BALF) was collected by flushing the right lobes as previously described (9). After red blood cell lysis, cells from BALF were cytospun onto glass slides and stained with May-Grunwald/Giemsa for further differential leukocyte counts. The left lung lobes were either stored at -80°C for later mRNA and protein analysis or fixed in 10% neutral buffered formalin for histological examination. Paraffin-embedded sections were stained with alcian blue/periodic acid-Schiff (AB-PAS) for assessment of hyperplasia of mucus-secreting goblet cells or with Hematoxylin and eosin (H&E) for determination of inflammation score as described previously (10, 11).

### Sample Preparation for Mass Spectrometry

Lung proteins were extracted by homogenizing in RIPA buffer (radioimmunoprecipitation assay buffer, Sigma-Aldrich) with 1% protease inhibitor (Cell Signaling Technology; Danvers, MA). Protein concentration in the supernatant was measured using the bicinchoninic acid (BCA) assay. An equal amount of proteins (200 µg) from each mouse in the same group was pooled, and 200 µg protein from each group was mixed with denaturation buffer to a final concentration of 6 M urea and 2 M thiourea. Denatured proteins were reduced using 10 mM dithiothreitol, alkylated using 20 mM iodoacetamide, and subsequently digested into peptides using 1:40 (enzyme: protein) ratio of Trypsin/Lys-C mix endoproteinase (mass spec grade, Promega) to activate Lys-C digestion. After Lys-C digestion, the solution was diluted below 1 M urea, 0.33 M thiourea by adding 20 mM tetraethylammonium bromide (pH 7.8) and incubated overnight to activate trypsin digestion. Peptide samples were desalted using Visiprep™ vacuum manifold (12-port, Sigma-Aldrich) coupled with Empore™ C18 Solid-phase extraction (SPE) cartridge (4 MM per 1 ml) according to the manufacturer’s instructions. The peptide eluents were quantified using the Invitrogen™ Qubit^®^ Protein Assay (Life Technologies Australia Pty Ltd), lyophilized and resuspended with MS loading buffer (2% acetonitrile, 0.1% trifluoroacetic acid). All resuspended samples were store at -80°C before mass spectrometry.

### Liquid Chromatography-Tandem Mass Spectrometry (LC-MS/MS) for Shotgun Proteomics

500 ng peptides were injected and separated prior to MS using a Dionex ultimate Nano/Cap 3500-RS LC system (Thermo Fisher Scientific) at a constant flow rate of 300 nL min^-1^ using an EASY-Spray PepMap C18 LC column (75 μm × 15 cm, C_18_, 2 μM, 100 Å, Thermo Fisher Scientific), with a 150-min gradient. Solvent A was water, 0.1% formic acid, and solvent B was acetonitrile, 0.1% formic acid; peptides were first injected with 2% solvent B for 6 min and eluted by a gradient from 2 to 35% solvent B from 6 to 126 min, then 35 to 90% solvent B over a further 0.1 min, the remaining peptides were washed away using 90% solvent B for 1.9 min followed by a 22 min equilibration step (2% solvent B) before LC returning to the starting conditions. Q-Exactive Plus High-Resolution Quadrupole-Orbitrap™ (Thermo fisher scientific) tandem mass spectrometry (MS/MS) was used for the discovery of MS assay. Precursor ions were measured with a full mass scan (400-2000 m/z, with a resolution of 70,000), an AGC target value at 1e6 and a maximum injection fill time is 50 ms. The product ions were fragmented with a normalized collision energy of 27.0 and measured at a resolution of 35,000 on the Orbitrap. AGC target value was set at 2e5 and the maximum injection fill time was 120 ms to control the correct ion population within the orbitrap. Dynamic exclusion was employed for 30 s. Each group was run duplicate for shotgun proteomics.

### Shotgun Proteomics for Protein Identification and Quantification

Shotgun proteomic raw data were analyzed to generate a protein/peptide identification report using the Sequest HT search engine in Thermo Proteome Discoverer (version 2.4.1.15) against all reviewed mouse entries (canonical only) in the Uniprot proteome database (downloaded May 20, 2020, with a total of 36,352 entries). The following search parameters were used: mass tolerances of precursor and product ions were 10 ppm and 0.02 Da, respectively; trypsin was designated as the digestion enzyme, and up to two missed cleavages were allowed; Protein confidence indicators were thresholded at ≤ 1% (strict) and ≤ 5% (relaxed) false discovery rate (FDR). Oxidation of methionine, deamidation of asparagine and glutamine, carbamidomethylation of cysteines, acetylation of lysine, methylation of lysine, phosphorylation of serine, threonine, and tyrosine, loss of methionine and loss + acetylation of methionine were set as dynamic modifications. Relative label-free protein quantification analysis was conducted by using the Minora algorithm in Thermo Proteome Discoverer (version 2.4.1.15). The following quantification parameters were used: the minimum trace length was 5; the maximum retention time difference of isotope pattern multiples was 0.2 min, and PSM verification confidence was high. Two replicates of OVA, OVA/FLU and OVA/FLU/DEX were grouped and compared with PBS control to calculate average expression fold changes for individual proteins. Significance assessment was performed using the individual proteins-based ANOVA method implemented in Proteome Discoverer 2.4 and multiple comparison adjustment of the p values was performed by Benjamini-Hochberg correction method (12, 13).

### Clustering Analysis for Heatmap Visualization

All identified proteins of lung tissue proteome were filtered based on the following rules: ≤ 1% FDR, ≥ 2 unique peptides, protein expression fold change (OVA, OVA/FLU, or OVA/FLU/DEX versus PBS control ≥ 2 or ≤ -2, p-value ≤ 0.05) to select proteins that were detected in high confidence and were significantly changed between mouse treatment groups. To prepare the data set for clustering analysis, the protein expression fold change value of all significantly changed proteins from each type of treated mice were used, while treated mice with no significant change for a particular protein were assigned as zero for that protein in the data set. For heatmap visualization, the data set was imported into TBtools (14) and transformed to Z-score scale for normalization. The clustering analysis was performed using the Euclidean complete method for distance and aggregation of all proteins.

### Ingenuity Pathway Analysis (IPA) Analysis

Based on the clustering analysis results, only proteins that over-expressed or under-expressed in OVA/FLU mice but not in OVA mice were included, and further analyzed using IPA to define the enriched protein signaling pathways and to predict upstream regulators. The analysis was performed by comparing all significantly changed proteins against known canonical pathways within the standard IPA library. A right-tailed Fisher’s exact test was used to calculate the significance of pathways and upstream regulator. The activation state of upstream regulator was analyzed based on the expression fold changes for individual proteins (asthmatic versus PBS). The major protein signal transduction networks that led to the immunopathogenesis of steroid resistant asthma exacerbation were reconstructed according to all IPA results as well as the literature searching.

### PRM Proteomic Analysis

Parallel reaction monitoring (PRM) proteomic is an ion monitoring technique based on high-resolution mass spectrometry which is more convenient in assay development for precise quantification of proteins and peptides of interest (15). Among all major protein components for the immunopathogenesis of asthma exacerbation, using label-free shotgun proteomic approach, STAT1 was found to be among the most highly relevant protein and was then validated by PRM mass spectrometry. Proteins extracted from PBS, OVA, OVA/FLU, and OVA/FLU/DEX were digested into peptides as described above (see protein extraction and sample preparation for mass spectrometry). Two standard peptides (K.LQHSLDTALR.R and R.SAPAAAIAAR.V) from the precursor of nerve growth factor (proNGF) were spiked into peptide samples at the optimized concentration (25 ftamol/μl) to obtain the relative quantification. 500 ng peptides were injected and separated before MS using a Dionex ultimate Nano/Cap 3500-RS LC system as described above in the section Liquid chromatography-tandem mass spectrometry (LC-MS/MS) for shotgun proteomics. A 70-min short gradient was used. Solvent A was water, 0.1% formic acid, and solvent B was acetonitrile, 0.1% formic acid; peptides were first injected with 2% solvent B for 5 min and eluted by a gradient from 2 to 40% solvent B from 5 to 45 min, then 40 to 95% solvent B over a further 2 min, the remaining peptides were washed away using 95% solvent B for 3 min followed by a 20 min equilibration step (2% solvent B) before LC returning to the starting conditions. A Q-Exactive Plus High-Resolution Quadrupole-Orbitrap™ (Thermo fisher scientific) tandem mass spectrometer (MS/MS) was used. PRM methods were optimized by screening all tryptic peptides for protein STAT1 according to mass state, charge state, and product ion peak quality. Two unique peptides, K.TLEELQDEYDFK.C and K.VMAAENIPENPLK.Y, with the highest product ion peak intensity and confidence were selected for final quantification of STAT1. Precursor ions were measured with a full mass scan (390-2000 m/z, with a resolution of 70,000), an AGC target value at 1e6 and a maximum injection fill time is 50 ms. The product ions were fragmented with stepped collision energy from 26.0 to 30.0 and measured at a resolution of 35,000 on the Orbitrap. AGC target value was set at 2e5 and maximum injection fill time was 128 ms to control for the correct ion population within the orbitrap. These raw data were analyzed using Skyline-daily software 64-bit, version 19.0.0.190 (MacCoss Lab, University of Washington, Seattle, WA). Quantification was performed by measuring the peak area for each product ion for each peptide, followed by normalizing according to the two standard peptides.

### Western Blotting

Lung protein was extracted as described above. Proteins were separated on stain-free gels (Bio-rad) and transferred onto polyvinylidene fluoride (PVDF) membranes and incubated with rabbit anti-STAT1 Ab (1:2000 dilution, Cell Signaling Technology) or with rabbit anti-phospho-STAT1 (Tyr701) Ab (1:2000 dilution, Cell Signaling Technology). Secondary Abs were goat anti-rabbit IgG (1:5000 dilution, Abcam). Anti-β-actin (1:2000 dilution, Biolegend) was incubated after stripping the STAT1 or P-STAT1 Ab. The relative amount of STAT1 and P-STAT1 protein was quantified by normalizing the amount of β-actin housekeeping protein using Image Lab software 6.1.

### Statistical Analysis

GraphPad Prism (Version 9.0, USA) was used for all data analysis. Lung function data were analyzed using two-way ANOVA, while all other forms of analysis were conducted using one-way ANOVA (plus Tukey *post hoc* test multi-comparison test). n = 6–8 mice/group, data represented as mean ± SEM. Differences will be considered statistically significant if p<0.05.

## Results

### Viral Infection Induced Severe Steroid-Resistant Asthma Exacerbation With Augmented AHR and Airway Inflammation

**Figure 1:**
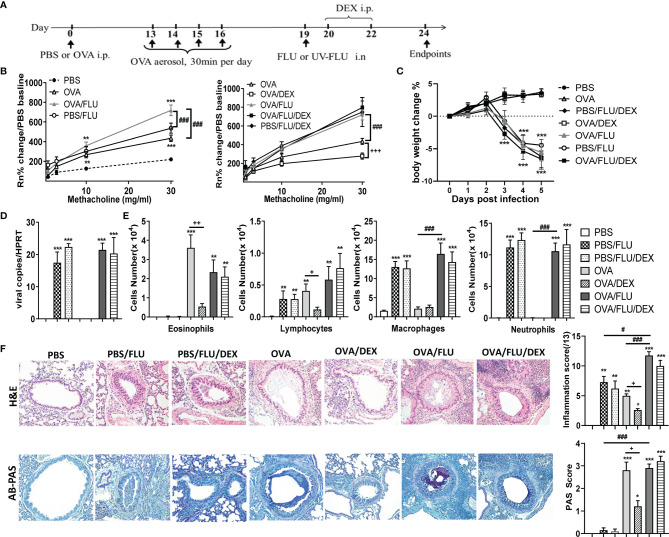
Viral infection of allergic asthmatic mice induces increased airway hyperresponsiveness (AHR) and inflammation which does not respond to dexamethasone (DEX) treatment. BALB/c mice were sensitized i.p. with OVA/PBS on day 0 and exposed to OVA aerosol on days 13–16. Some mice were inoculated with FLU on day 19 and/or treated with DEX on days 20 and 22, and lung function and airway inflammation were assessed on day 24. **(A)** experimental design; **(B)** AHR; **(C)** body weight changes over 0 dpi; **(D)** viral load in lung tissue assessed by qPCR; **(E)** differential inflammatory cell counts for eosinophils, lymphocytes, macrophages, and neutrophils in BALF; **(F)** histologic examination of lungs stained with H& E for assessment of inflammatory score or AB-PAS for assessment of mucus-secreting goblet cells hyperplasia. All images were randomly selected using a 20 × magnification lens. Data are presented as mean ± SEM (n = 6-8 mice/group) and are representative of three independent experiments. *Designates significant differences compared to PBS group (*p < 0.05, **p < 0.01, ***p < 0.001); #Designates significant differences compared to OVA/FLU treated group (#p < 0.05, ###p < 0.001); +Designates significant differences compared to DEX treated groups (+p < 0.05, ++p < 0.01, +++p < 0.001).

The establishment of an *in vivo* model of asthma exacerbation was displayed in **Figure 1A**. Mice treated with OVA alongside FLU infection (OVA/FLU) exhibited dramatically exacerbated AHR compared to OVA alone or the control PBS/FLU group (**Figure 1B**). FLU infections caused significant body weight loss, and viral replication was not affected by the allergic condition (**Figures 1C, D**). OVA and PBS/FLU groups displayed eosinophilic/lymphocytic or neutrophilic/macrophage inflammation, respectively, OVA/FLU groups exhibited increased levels of the four major inflammatory cells in BALF (**Figure 1E**). Histological analysis revealed increased inflammatory foci in the parenchyma and airway in the OVA/FLU group compared to PBS/FLU or OVA groups (**Figure 1F**). Additionally, OVA/FLU group showed no significant reduction in Th2 cytokine levels compared to OVA (**Figures S1A–E**). IFN-α, IFN-β, IFN-γ, TNF and IL-1β were significantly elevated in PBS/FLU but not OVA groups and were further increased in OVA/FLU mice (**Figures S1F–J**).

DEX treatment of the OVA group significantly suppressed AHR, decreased the infiltration of eosinophils and lymphocytes, goblet cells hyperplasia (**Figures 1B, E, F**), and transcription of Th2 cytokines (Figure S.1A-E). By contrast, DEX had no effect on these factors in the PBS/FLU or OVA/FLU groups (**Figures 1B, E, F**). DEX did not prevent weight loss or viral replication (**Figures 1C, D**) and had no effect on the transcript levels of IL-4, IL-5, Eotaxins, IFN-α/β/γ, TNF, or IL-1β, except for IL-13 in OVA/FLU/DEX group, and these factors are not affected in PBS/FLU/DEX group (**Figures S1A–J**). These data describe a unique mouse model of FLU-induced asthma exacerbation that is steroid-resistant with a mixed inflammatory response (cells and factors) and steroid-resistant AHR on the background of pathogenic type-2 inflammation.

### Profiling of the Lung Tissue Proteome in Allergic Asthma and Asthma Exacerbation

**Figure 2:**
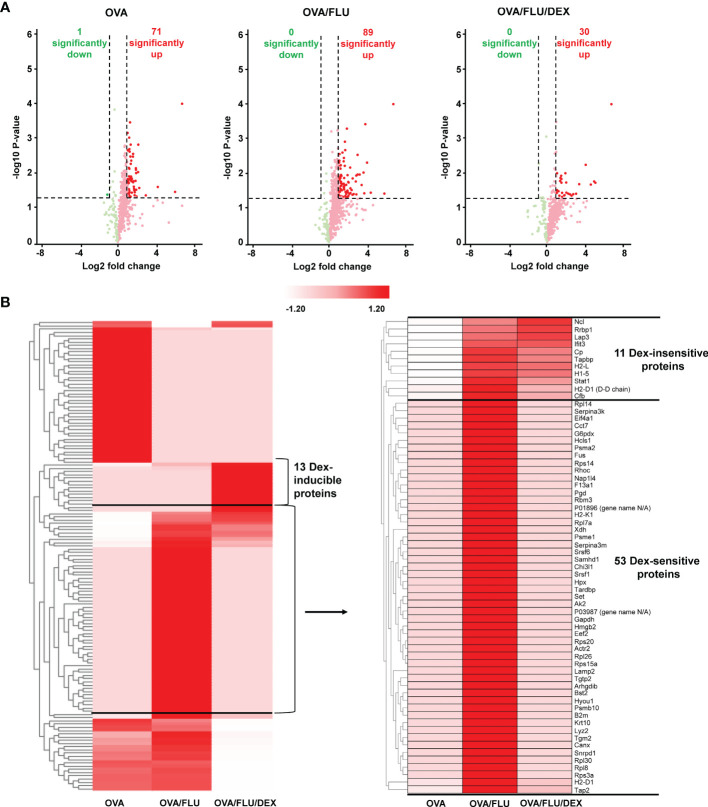
Profiling the mouse lung tissue proteome in allergic asthma and steroid-resistant asthma exacerbation. All samples were analyzed in biological duplicates and data presented are compared to PBS control. **(A)** Volcano plots represent protein changes in the lungs of all asthmatic mice (OVA or OVA/FLU or OVA/FLU/DEX). The x and y axes are in log scale. Dark red dots indicate significantly upregulated proteins whereas dark green dots indicate significantly downregulated proteins (threshold: protein fold change (asthmatic versus PBS ≥ 2 or ≤ -2, p-value ≤ 0.05). **(B)** Heatmap depicting expression of the significantly changed proteins in asthmatic mouse lung tissue compared to PBS controls (asthmatic versus PBS ≥ 2 or ≤ -2, p-value ≤ 0.05). Clustering analysis using Z-score for row scale normalization and Euclidean method for distance and aggregation. The heatmap shows a segment of proteins over-expressed exclusively in OVA/FLU/DEX mice (DEX inducible proteins) and a segment of proteins over-expressed in OVA/FLU treated mice but under-expressed in OVA mice. One cluster of proteins was also found highly expressed in OVA/FLU/DEX treated mice (DEX-insensitive) while another cluster of proteins was not (DEX-sensitive).

Next, we profiled the whole lung tissue proteome in three groups of asthmatic mice (OVA, OVA/FLU and OVA/FLU/DEX) and control mice (PBS) to investigate the mechanisms of FLU induced exacerbations (5 dpi). LC-MS/MS analysis identified 3,466 unique peptides corresponding to 1,221 individual proteins in OVA group (**Table S1**). Among them, 590 proteins were identified in high confidence (**Table S2**), with 71 significantly upregulated and 1 significantly downregulated by at least 2-fold (OVA verses PBS) (**Figure 2A** and **Table S3**). 3,540 unique peptides corresponding to 1,237 individual proteins were identified in the OVA/FLU group (**Table S1**), with 623 identified in high confidence (**Table S2**), among which 89 were significantly upregulated at least 2-fold but there were no downregulated proteins within this threshold (OVA/FLU verses PBS) (**Figure 2A** and **Table S4**). 3,169 unique peptides corresponding to 1,156 individual proteins were identified in the OVA/FLU/DEX group (**Table S1**); among which, 560 were identified in high confidence (**Table S2**), with 30 significantly up-regulated at least 2-fold and no downregulated proteins within this threshold (OVA/FLU/DEX verses PBS) (**Figure 2A** and **Table S5**).

For identification of proteins of interest, hierarchical clustering analysis was firstly performed on the data set of significantly changed proteins in asthmatic mice compared with PBS. A segment containing 64 proteins was found to be specifically highly expressed in OVA/FLU compared to OVA (**Figure 2B** and **Table S6**). Among them, 53 proteins were reduced to baseline after DEX treatment (DEX-sensitive), and 11 were consistently upregulated after DEX treatment (DEX-insensitive), which include interferon-induced protein with tetratricopeptide repeats-3 (IFIT3), tapasin (Tapbp), H-2 class I histocompatibility antigens (H2-L/H2-D1), and STAT1 (**Figure 2B** and **Table S6-1**). In addition, 13 proteins were elevated uniquely in DEX group (**Table S6-2**).

### Integrated Proteomic Pathway Analysis Reveals STAT1 as a Key Regulator of Asthma Exacerbation

**Figure 3:**
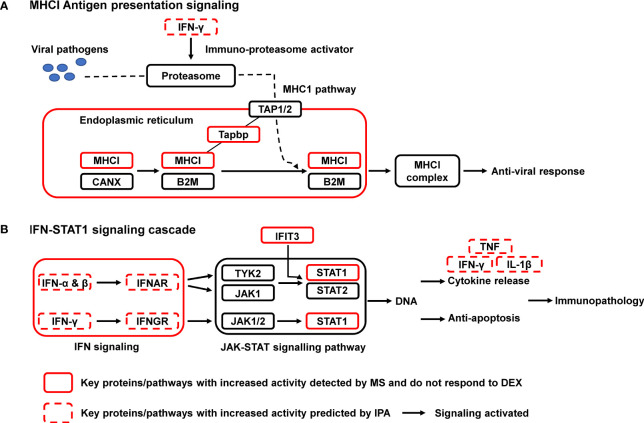
Signaling pathway enrichment in a mouse steroid-resistant asthma exacerbation model. The reconstruction of protein networks was based on Ingenuity Pathway Analysis (IPA) and literature searching. The most regulated signal transduction pathways and the major changes in protein components of these pathways are displayed. **(A)** MHCI Antigen presentation signaling; **(B)** IFN-STAT1 signaling cascade. MHC I, class I major histocompatibility complex; B2M, β2 macroglobulin; CANX, chaperone calnexin; TAP, transporters associated with antigen processing; Tapbp, tapasin, TAP binding protein; IFN, interferon; JAK, Janus tyrosine kinase; STAT, signal transducer and activator of transcription; IFNAR/IFNGR, interferon receptor. IFIT3, interferon induced protein with tetratricopeptide repeats 3; TNF, tumor necrosis factor.

All 64 significantly upregulated proteins in OVA/FLU mice were submitted to IPA for pathway analysis, which were potentially involved in 40 signaling pathways. Two of the most relevant pathways related to the immunopathology of exacerbation were MHC-I and IFN signaling pathways (**Table S7**). IPA analysis further revealed that the activities of 74 upstream regulators were significantly changed, including the significant activation of IFN-α/β/γ, and TNF (**Table S8**). By combining IPA analysis and literature search, a molecular blueprint of the major dysregulated signaling pathways involved in FLU-induced asthma exacerbation was created; MHC-I and IFN-STAT1 signaling are particularly interesting as they are known to be involved in antiviral responses and immunopathology (**Figures 3A, B**). Indeed, among those proteins upregulated in OVA/FLU group, Tapbp, H2-L (MHC-I molecule), IFIT3, and STAT1 were steroid resistant and implicated in MHC-I and IFN-STAT1 signaling (**Table S6-1**). Interestingly, IFIT3 is embedded in the STAT1-regulated signaling network (16), and label-free quantification revealed STAT1 was among the top upregulated (53-fold) proteins in OVA/FLU group compared to control, indicating that STAT1 might be the most important functional factor in these pathways. Thus, its protein expression was further validated by Parallel Reaction Monitoring (PRM) mass spectrometry and western blotting.

### PRM and Western Blot Validation of STAT1 Expression in Asthma Exacerbation

**Figure 4:**
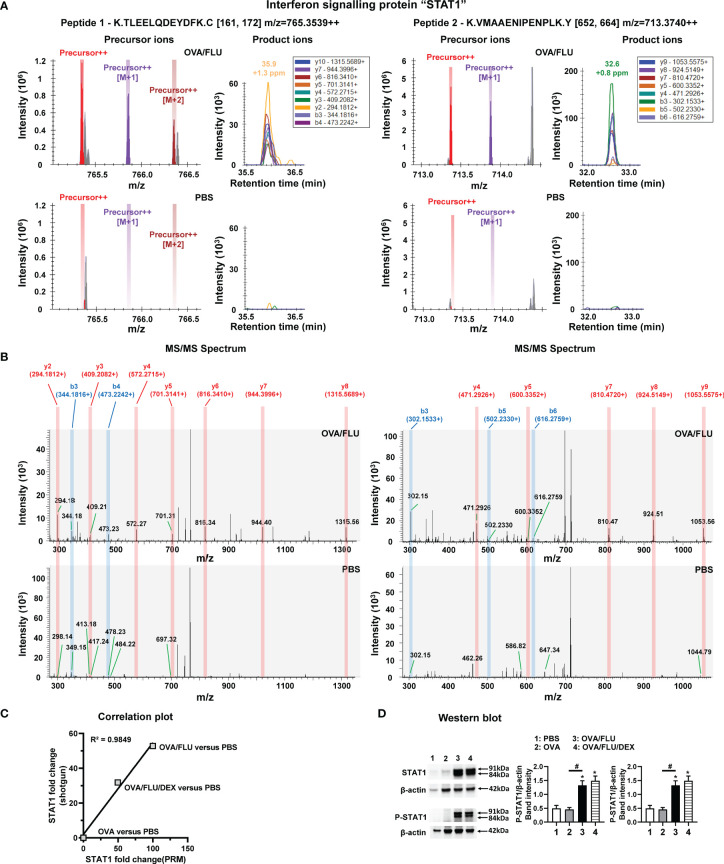
Parallel Reaction Monitoring (PRM) mass spectrometry and western blot of STAT1 protein expression. PRM mass spectrometry detected and quantified two unique peptides of STAT1, K.TLEELQDEYDFK.C (peptide 1) and K.VMAAENIPENPLK.Y (peptide 2), in OVA/FLU and OVA/FLU/DEX but not OVA or PBS groups (OVA/FLU versus PBS is shown as an example). **(A)** A serial precursor ions (each color bar represents one precursor ion), as well as their corresponding y and b product ions (each color peak represents one product ion) for both peptides, were accurately detected in the OVA/FLU but not PBS group. **(B)** MS/MS spectrum for both peptides validated that all y (labeled in red) and b (labeled in blue) product ions for both peptides of protein were accurately detected in the OVA/FLU but not in PBS group. **(C)** The correlation plot represents the quantification results of STAT1 (OVA or OVA/FLU OVA/FLU/DEX or versus PBS) obtained between shotgun label-free proteomic and PRM proteomic. The coefficient of linear regression (coefficient of determination R^2^) is 0.9849. **(D)** Western blot of the two isoforms of STAT1 (Stat1α, Stat1β) and the phosphorylated STAT1 (P-STAT1, on site Tyr 701) (phospho-Stat1α/phospho-Stat1β). β-actin (42 kDa) was used as a loading control. The relative quantification of STAT1 was performed using Image Lab software 6.1. Results are representative of three independent experiments. *Designates significant differences compared to PBS group (*p < 0.05); #Designates significant differences compared to OVA/FLU treated group (^#^p < 0.05).

The validation of STAT1 was firstly conducted by PRM. OVA/FLU versus PBS groups are shown as an example for successful PRM quantification of protein STAT1, all precursor ions, product ions and MS/MS spectrum for two unique peptides K.TLEELQDEYDFK.C and K.VMAAENIPENPLK.Y of STAT1 were detected and quantified in OVA/FLU group but not in PBS control group (**Figures 4A, B**). All quantitative product ions in other groups are presented in **Table S9**. The correlation curves were generated between protein level changes observed by shotgun label-free proteomic and PRM quantification (**Figure 4C** and **Table S10**), where the Pearson correlation coefficient (coefficient of determination, R^2^) was 0.9849, indicating a “high” correspondence in results obtained by the two quantification approaches. To confirm the mass spectrometry results, western blot analysis was employed. Both Stat1α/Stat1β (91 and 84 kDa) and phosphorylated STAT1(P-STAT1, site Tyr 701), were significantly upregulated in OVA/FLU and OVA/FLU/DEX groups compared to OVA and PBS groups (**Figures 4D** and **S2A**). Therefore, our proteomic analysis output was deemed reliable.

### STAT1 Inhibition Significantly Suppresses AHR and Inflammation in the Asthma Exacerbation Model

**Figure 5:**
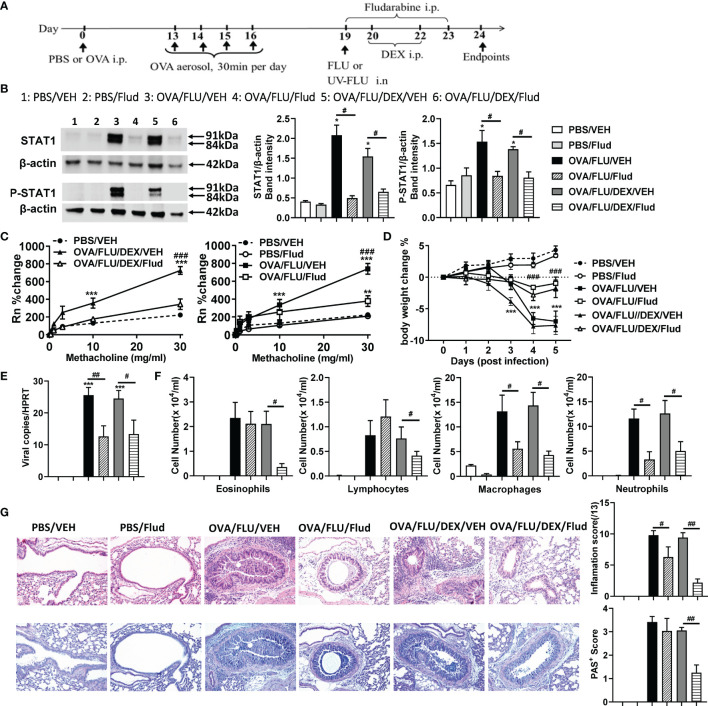
Fludarabine treatment inhibits STAT1 production and suppresses AHR and inflammation in the asthma exacerbation model. BALB/c mice were sensitized i.p. with OVA or PBS on day 0 and exposed to OVA aerosol on days 13–16. Some mice were inoculated with FLU on day 19, treated with Dex on days 20 and 22, or injected with Fludarabine from day 19-23 as indicated. Lung function and airway inflammation were assessed on day 24. **(A)** experimental design; **(B)** Western blot analysis confirmed the efficiency of Fludarabine inhibiting STAT1, using anti-STAT1 Ab (Stat1α, Stat1β) and anti-phospho-STAT1 (Tyr701) Ab. β-actin (42 kDa) was used as a loading control. The relative quantification of STAT1 proteins were performed using Image Lab software 6.1. **(C)** AHR; **(D)** body weight changes over 0 dpi; **(E)** viral load in lung tissue; **(F)** differential inflammatory cell counts for neutrophils, lymphocytes, macrophages, and eosinophils in BALF; **(G)** histologic examination of lung tissue stained with H&E or AB-PAS for assessment of inflammatory score or hyperplasia of mucus-secreting goblet cells, respectively. All histological images were randomly selected using a 20 × magnification lens. Data are presented as mean ± SEM (n = 6-8 mice/group) and are representative of three independent experiments. *Designates significant differences from PBS-treated controls (*p < 0.05, **p < 0.01, ***p < 0.001). #Designates significant differences between Flud-treated and isotype-treated groups (#p < 0.05, ##p < 0.01, ###p < 0.001).

To validate our finding, we inhibited STAT1 with Fludarabine (Flud). The experimental design is displayed in **Figure 5A**. OVA/FLU mice treated with Flud exhibited significantly reduced expression of both isotypes of STAT1 and P-STAT1 (**Figures 5B** and **S2B**). Although there was no significant effect on the infiltration of eosinophils, lymphocytes or on goblet cells hyperplasia, Flud-treatment was accompanied by significantly reduced macrophages and neutrophils, attenuated AHR, prevented body weight loss, and suppression of FLU viral-replication (**Figures 5C**–**F**); it also decreased inflammation scores compared to vehicle control (**Figure 5G**). The transcript levels of IFN-α/β/γ, TNF, and IL-1β but not Th2 cytokines were suppressed (**Figures S3A–J**). Interestingly, with DEX and Flud combined-treatment, these not-affected hallmarks (eosinophils, lymphocytes, and goblet cells hyperplasia and expression of Type-2 molecules) were significantly reduced compared to the non-DEX treated group (i.e., OVA/FLU, or OVA/FLU/Flud), accompanied by completely suppressed AHR and decreased tissue inflammation hallmarks (**Figures 5C**–**G** and **S3A–J**). These data indicate the important effect of inhibiting STAT1 with Flud on controlling viral-induced immunopathology, and in resolving the problem of steroid resistance in our asthma exacerbation model.

## Discussion

To understand the pathogenesis of asthma exacerbation, we established a FLU-induced exacerbation mouse model and profiled the lung proteome using liquid chromatography coupled with tandem mass spectrometry (LC-MS/MS) and multiple bioinformatic software. We identified the important roles of MHC-I antigen presenting and IFN-STAT1 signaling pathways in triggering asthma exacerbation. Furthermore, we have identified STAT1 as a potential therapeutic target for reducing immunopathology and restoring steroid sensitivity during FLU-triggered exacerbation.

Asthma is a complex immune disorder with two major immune phenotypes, Th2 high and Th2 low airway inflammation (17). Our asthma model (OVA group) exhibited typical Th2 high inflammation, in which the hallmarks of inflammation can be reduced by DEX treatment. Our asthma exacerbation model (OVA/FLU group) exhibited Th2 low inflammation, with dramatically increased neutrophils and macrophages in the lung and a cytokine expression pattern of low Th2 combined with high non-Th2 cytokines, which were largely resistant to DEX treatment. These results are consistent with previously reported findings that the major cellular feature of Th2 low airway inflammation involves innate immune cells infiltration (neutrophils and macrophages) (18), which are closely related to the steroid resistance of asthmatic disease (19–21).

Despite evidence highlighting the innate immune cells as a hallmark feature of asthma exacerbation, the molecular mechanisms underlying the infiltrates remain poorly understood. Using a shotgun proteomics assay we analyzed the protein expression profiles of lung tissue from mice during asthma exacerbation. As far as we have learned from the literature, this is one of the most comprehensive proteomics investigations for studying the entire proteome of influenza infection and asthma exacerbations in a single study using mouse model systems. We revealed not only the protein profiles of the asthma exacerbation, but also the mechanism of steroid resistance. Besides the DEX-sensitive and DEX-insensitive proteins, a small cluster of DEX-inducible proteins (Chil4, ITGB2, and Fcer1g with high fold change) were also identified. These factors have regulatory effects on the activities of eosinophils, NK cells, T cells, neutrophils, and mast cells (22–24), indicating that DEX may also play a role in promoting leukocytes migration.

Of particular interest, our IPA results for the first time showed that two of the most upregulated pathways in FLU-induced asthma exacerbation (compared to allergic asthma without infection) were MHC-I antigen-presenting and IFN signaling pathways. Given that these pathways are important in the regulation of the innate immune response (25–28), our results provide evidence supporting the key role of MHC-I and IFN signaling pathways in triggering immunopathogenesis of asthma exacerbation. Many studies have reported that suppression of MHC-I and IFN signaling downregulates inflammation in numerous diseases (e.g., infectious disease, cancer, autoimmune disease) (29–34), highlighting the potential to treat asthma exacerbation by targeting these pathways.

Among all proteins identified within the MHC-I and IFN pathways, STAT1 was the most attractive candidate for therapeutic intervention. STAT1 was not only one of the most significantly upregulated protein, but also insensitive to DEX-treatment. As a central mediator of IFN signaling (35), its activation enables the transcription of IFN-stimulated genes involved in a variety of cellular activities, e.g., apoptosis, regulation of inflammation and immunosurveillance (36–39). Downregulation of STAT1 has been shown to inhibit inflammation in respiratory diseases, e.g., chronic obstructive pulmonary disease (40), and potentially to treat severe COVID-19 disease (41).

Further, we tested the effect of targeting STAT1 on asthma exacerbation by using Fludarabine, a pharmaceutical drug that decreases the activation of STAT1 and is currently used clinically to treat certain cancers (42–44). We observed that treatment with Fludarabine together with dexamethasone greatly decreased the key cellular features of asthma exacerbation including innate inflammatory cells infiltration into the lung and goblet cells hyperplasia. Combination therapy also decreased the levels of Th2 and innate inflammatory cytokines, and attenuated AHR and tissue inflammation. Our findings highlight that Fludarabine has the potential to restore steroid sensitivity in asthma during exacerbation, by attenuating the exaggerated inflammatory response and immunopathology characteristic of disease. To date, although many studies have spent significant efforts and costs on discovering novel drugs that target pro-inflammatory factor(s) to prevent or reduce inflammation associated with asthma exacerbation, only a few have been effective (45). Notably, type 2 biologics targeting Th2 high asthma are relatively effective, however, individuals still exacerbate even with these treatments. On the other hand, further effective treatments for Th2 low asthma are still required. Here, our study suggests that the combination of clinical available drugs, Fludarabine and DEX, could be directly added into the existing treatment protocols for steroid-resistant asthma exacerbation induced by FLU. Future studies are necessary to perform both the pharmacokinetics and pharmacodynamics analyses for optimizing the drug administration (e.g. dose and frequency), as both drugs can cause side-effects when used long-term or at high doses (46).

We also observed that Fludarabine treatment significantly reduced viral load in FLU-infected mice and prevented infection-mediated body weight loss. Further, the elevated anti-viral IFNα/β transcription levels following FLU infection were all reduced by Fludarabine treatment. Although STAT1 deficient mice has shown decreased anti-viral responses (47, 48), our data is consistent with the previously reported finding that Fludarabine has an anti-viral effect on influenza virus (49). Several studies have reported that Fludarabine has anti-viral activity against many RNA viruses including zika virus, severe-fever-with-thrombocytopenia-syndrome-virus and enterovirus (50). This may be because Fludarabine can compete with natural nucleotides for incorporation into viral RNA (vRNA), thus interrupting vRNA synthesis (50). This evidence highlights that Fludarabine has the potential to be re-purposed as an anti-viral drug for targeting RNA viruses, however, the mechanism and the clinical significance need to be further evaluated.

Here, we provide a detailed proteomic profile, identifying dysregulated proteins associated with the pathogenesis and disease progression of FLU-induced exacerbation of asthma. More importantly, as far as we are aware, this is the first study that highlights the potential clinical utility of using Fludarabine in combination with DEX, for the treatment of asthma exacerbation. Of note, the protein dataset that we presented in this study is publicly accessible and can be employed for future investigations of the pathological processes of asthma exacerbation.

## Data Availability Statement

The proteomic raw data has been deposited in the Mass Spectrometry Interactive Virtual Environment (MassIVE) database with the dataset identifier: MSV000088182, and are publicly accessible by the link: http://massive.ucsd.edu/ProteoSAFe/status.jsp?task=50be5623fc2f4702baf623ddcd5d93f7.

## Ethics Statement

The animal study was reviewed and approved by Animal Care and Ethics Committee in the University of Newcastle.

## Author Contributions

XML and XL contributed to animal model development, data curation and analysis, and writing the original draft. LC, AH, and KA contributed to validation and editing draft. CL, KL, and IB contributed to the draft reviewing and supplied virus resources. PF and MY supervised this study and edited the final manuscript. All authors contributed to the article and approved the submitted version.

## Conflict of Interest

The authors declare that the research was conducted in the absence of any commercial or financial relationships that could be construed as a potential conflict of interest.

## Publisher’s Note

All claims expressed in this article are solely those of the authors and do not necessarily represent those of their affiliated organizations, or those of the publisher, the editors and the reviewers. Any product that may be evaluated in this article, or claim that may be made by its manufacturer, is not guaranteed or endorsed by the publisher.

## Abbreviations

AHR, airway hyper-responsiveness; AB-PAS, alcian blue/periodic acid-Schiff; BALF, bronchoalveolar lavage fluid; DEX, dexamethasone; dpi, days post infection; FLU, influenza virus; Flud, Fludarabine; IFNs, interferons; i.n., intranasally; i.p., intraperitoneally; IPA, Ingenuity Pathway Analysis; LC-MS/MS, liquid chromatography coupled with tandem mass spectrometry; MHC-I, major histocompatibility complex class I; OVA, ovalbumin; PRM, Parallel Reaction Monitoring proteomic; PBS, phosphate-buffered saline; RV, rhinovirus; RSV, respiratory syncytial virus; STAT1, signal-transducer-and-activator-of-transcription-1; Th, CD4^+^ T-helper cells; TNF, tumor necrosis factor.

## References

[1] National Heart, Lung, and Blood Institute National Asthma Education and Prevention Program. Expert Panel Report 2: Guidelines for the Diagnosis and Management of Asthma. Bethesda MD: US Department of Health and Human Services, National Institutes of Health (1997).

[2] Lemanske RF Jr Mauger DT Sorkness CA Jackson DJ Boehmer SJ Martinez FD. Step-Up Therapy for Children With Uncontrolled Asthma Receiving Inhaled Corticosteroids. N Engl J Med (2010) 362(11):975–85. doi: 10.1056/NEJMoa1001278 PMC2989902 20197425

[3] Network GA. The Global Asthma Report. Auckland, New Zealand: Global Asthma Network (2018).

[4] Jartti T Bønnelykke K Elenius V Feleszko W. Role of Viruses in Asthma. Semin Immunopathol (2020) 42(1):61–74. doi: 10.1007/s00281-020-00781-5 31989228 PMC7066101

[5] Zhu J. T Helper 2 (Th2) Cell Differentiation, Type 2 Innate Lymphoid Cell (ILC2) Development and Regulation of Interleukin-4 (IL-4) and IL-13 Production. Cytokine (2015) 75(1):14–24. doi: 10.1016/j.cyto.2015.05.010 26044597 PMC4532589

[6] Merckx J Ducharme FM Martineau C Zemek R Gravel J Chalut D. Respiratory Viruses and Treatment Failure in Children With Asthma Exacerbation. Pediatrics (2018) 142(1):e20174105. doi: 10.1542/peds.2017-4105 29866794

[7] Zhao B Shan J Xiong R Xu K Li B. H1N1 Virus Production and Infection. Bio-Protocol (2018) 8(20):e3062–2. doi: 10.21769/BioProtoc.3062 PMC8342098 34532527

[8] Baer A Kehn-Hall K. Viral Concentration Determination Through Plaque Assays: Using Traditional and Novel Overlay Systems. J Vis Exp (2014) 93:e52065. doi: 10.3791/52065 PMC4255882 25407402

[9] Liu X Nguyen TH Sokulsky L Li X Garcia Netto K Hsu AC. IL-17A Is a Common and Critical Driver of Impaired Lung Function and Immunopathology Induced by Influenza Virus, Rhinovirus and Respiratory Syncytial Virus. Respirology (2021) 26(11):1049–59. doi: 10.1111/resp.14141 34472161

[10] Horvat JC Beagley KW Wade MA Preston JA Hansbro NG Hickey DK. Neonatal Chlamydial Infection Induces Mixed T-Cell Responses That Drive Allergic Airway Disease. Am J Respir Crit Care Med (2007) 176(6):556–64. doi: 10.1164/rccm.200607-1005OC 17600276

[11] Temelkovski J Hogan SP Shepherd DP Foster PS Kumar RK. An Improved Murine Model of Asthma: Selective Airway Inflammation, Epithelial Lesions and Increased Methacholine Responsiveness Following Chronic Exposure to Aerosolised Allergen. Thorax (1998) 53(10):849–56. doi: 10.1136/thx.53.10.849 PMC1745083 10193371

[12] Federspiel JD Greco TM Lum KK Cristea IM. Hdac4 Interactions in Huntington's Disease Viewed Through the Prism of Multiomics. Mol Cell Proteomics (2019) 18(8):S92–S113. doi: 10.1074/mcp.RA118.001253 31040226 PMC6692770

[13] Thermofisher. Proteome Discoverer User Guide Software Version 2.2 (2017). Available at: https://assets.thermofisher.com/TFS-Assets/CMD/manuals/Man-XCALI-97808-Proteome-Discoverer-User-ManXCALI97808-EN.pdf.

[14] Chen C Chen H Zhang Y Thomas HR Frank MH He Y. TBtools: An Integrative Toolkit Developed for Interactive Analyses of Big Biological Data. Mol Plant (2020) 13(8):1194–202. doi: 10.1016/j.molp.2020.06.009 32585190

[15] Park J Oh HJ Han D Wang JI Park IA Ryu HS. Parallel Reaction Monitoring-Mass Spectrometry (PRM-MS)-Based Targeted Proteomic Surrogates for Intrinsic Subtypes in Breast Cancer: Comparative Analysis With Immunohistochemical Phenotypes. J Proteome Res (2020) 19(7):2643–53. doi: 10.1021/acs.jproteome.9b00490 31755719

[16] Yang Y Zhou Y Hou J Bai C Li Z Fan J. Hepatic IFIT3 Predicts Interferon-A Therapeutic Response in Patients of Hepatocellular Carcinoma. Hepatology (2017) 66(1):152–66. doi: 10.1002/hep.29156 28295457

[17] Wenzel SE. Asthma Phenotypes: The Evolution From Clinical to Molecular Approaches. Nat Med (2012) 18(5):716–25. doi: 10.1038/nm.2678 22561835

[18] Duvall MG Krishnamoorthy N Levy BD. Non-Type 2 Inflammation in Severe Asthma Is Propelled by Neutrophil Cytoplasts and Maintained by Defective Resolution. Allergol Int (2019) 68(2):143–9. doi: 10.1016/j.alit.2018.11.006 30573389

[19] Goleva E Hauk PJ Hall CF Liu AH Riches DW Martin RJ. Corticosteroid-Resistant Asthma Is Associated With Classical Antimicrobial Activation of Airway Macrophages. J Allergy Clin Immunol (2008) 122(3):550–559.e3. doi: 10.1016/j.jaci.2008.07.007 18774390 PMC3930345

[20] Nabe T. Steroid-Resistant Asthma and Neutrophils. Biol Pharm Bull (2020) 43(1):31–5. doi: 10.1248/bpb.b19-00095 31902928

[21] Wang M Gao P Wu X Chen Y Feng Y Yang Q. Impaired Anti-Inflammatory Action of Glucocorticoid in Neutrophil From Patients With Steroid-Resistant Asthma. Respir Res (2016) 17(1):153. doi: 10.1186/s12931-016-0462-0 27852250 PMC5112750

[22] Webb DC McKenzie AN Foster PS. Expression of the Ym2 Lectin-Binding Protein Is Dependent on Interleukin (IL)-4 and IL-13 Signal Transduction: Identification of a Novel Allergy-Associated Protein. J Biol Chem (2001) 276(45):41969–76. doi: 10.1074/jbc.M106223200 11553626

[23] Crozat K Eidenschenk C Jaeger BN Krebs P Guia S Beutler B. Impact of β2 Integrin Deficiency on Mouse Natural Killer Cell Development and Function. Blood (2011) 117(10):2874–82. doi: 10.1182/blood-2010-10-315457 21239699

[24] Sakurai D Yamasaki S Arase K Park SY Arase H Konno A. Fcєrig-ITAM Is Differentially Required for Mast Cell Function *In Vivo* . J Immunol (2004) 172(4):2374–81. doi: 10.4049/jimmunol.172.4.2374 14764707

[25] Cresswell P Ackerman AL Giodini A Peaper DR Wearsch PA. Mechanisms of MHC Class I-Restricted Antigen Processing and Cross-Presentation. Immunol Rev (2005) 207(1):145–57. doi: 10.1111/j.0105-2896.2005.00316.x 16181333

[26] Kwaa AKR Talana CAG Blankson JN. Interferon Alpha Enhances NK Cell Function and the Suppressive Capacity of HIV-Specific CD8(+) T Cells. J Virol (2019) 93(3):e01541–18. doi: 10.1128/JVI.01541-18 PMC6340025 30404799

[27] Kopitar-Jerala N. The Role of Interferons in Inflammation and Inflammasome Activation. Front Immunol (2017) 8:873. doi: 10.3389/fimmu.2017.00873 28791024 PMC5525294

[28] Sambhara SR Miller RG. Programmed Cell Death of T Cells Signaled by the T Cell Receptor and the Alpha 3 Domain of Class I MHC. Science (1991) 252(5011):1424–7. doi: 10.1126/science.1828618 1828618

[29] Makris S Paulsen M Johansson C. Type I Interferons as Regulators of Lung Inflammation. Front Immunol (2017) 8:259. doi: 10.3389/fimmu.2017.00259 28344581 PMC5344902

[30] Goritzka M Durant LR Pereira C Salek-Ardakani S Openshaw PJ Johansson C. Alpha/beta Interferon Receptor Signaling Amplifies Early Proinflammatory Cytokine Production in the Lung During Respiratory Syncytial Virus Infection. J Virol (2014) 88(11):6128–36. doi: 10.1128/JVI.00333-14 PMC4093897 24648449

[31] Crouse J Kalinke U Oxenius A. Regulation of Antiviral T Cell Responses by Type I Interferons. Nat Rev Immunol (2015) 15(4):231–42. doi: 10.1038/nri3806 25790790

[32] Nguyen TH Maltby S Tay HL Eyers F Foster PS Yang M. Identification of IFN-G and IL-27 as Critical Regulators of Respiratory Syncytial Virus–Induced Exacerbation of Allergic Airways Disease in a Mouse Model. J Immunol (2018) 200(1):237–47. doi: 10.4049/jimmunol.1601950 29167232

[33] Yang J Yi Q. Killing Tumor Cells Through Their Surface β2-Microglobulin or Major Histocompatibility Complex Class I Molecules. Cancer (2010) 116(7):1638–45. doi: 10.1002/cncr.24953 PMC2847067 20143445

[34] Matzaraki V Kumar V Wijmenga C Zhernakova A. The MHC Locus and Genetic Susceptibility to Autoimmune and Infectious Diseases. Genome Biol (2017) 18(1):76. doi: 10.1186/s13059-017-1207-1 28449694 PMC5406920

[35] Chen K Liu J Liu S Xia M Zhang X Han D. Methyltransferase SETD2-Mediated Methylation of STAT1 is Critical for Interferon Antiviral Activity. Cell (2017) 170(3):492–506. doi: 10.1016/j.cell.2017.06.042 28753426

[36] Schindler C Levy DE Decker T. JAK-STAT Signaling: From Interferons to Cytokines. J Biol Chem (2007) 282(28):20059–63. doi: 10.1074/jbc.R700016200 17502367

[37] Lin CF Lin CM Lee KY Wu SY Feng PH Chen KY. Escape From IFN-γ-Dependent Immunosurveillance in Tumorigenesis. J BioMed Sci (2017) 24(1):10. doi: 10.1186/s12929-017-0317-0 28143527 PMC5286687

[38] Loh CY Arya A Naema AF Wong WF Sethi G Looi CY. Signal Transducer and Activator of Transcription (STATs) Proteins in Cancer and Inflammation: Functions and Therapeutic Implication. Front Oncol (2019) 9:48. doi: 10.3389/fonc.2019.00048 30847297 PMC6393348

[39] Zhang Y Mao D Roswit WT Jin X Patel AC Patel DA. PARP9-DTX3L Ubiquitin Ligase Targets Host Histone H2BJ and Viral 3c Protease to Enhance Interferon Signaling and Control Viral Infection. Nat Immunol (2015) 16(12):1215–27. doi: 10.1038/ni.3279 PMC4653074 26479788

[40] Xander N Reddy Vari H Eskandar R Li W Bolla S Marchetti N. Rhinovirus-Induced SIRT-1 *via* TLR2 Regulates Subsequent Type I and Type III IFN Responses in Airway Epithelial Cells. J Immunol (2019) 203(9):2508–19. doi: 10.4049/jimmunol.1900165 PMC6810856 31548332

[41] Luo W Li YX Jiang LJ Chen Q Wang T Ye DW. Targeting JAK-STAT Signaling to Control Cytokine Release Syndrome in COVID-19. Trends Pharmacol Sci (2020) 41(8):531–43. doi: 10.1016/j.tips.2020.06.007 PMC7298494 32580895

[42] Frank DA Mahajan S Ritz J. Fludarabine-Induced Immunosuppression Is Associated With Inhibition of STAT1 Signaling. Nat Med (1999) 5(4):444–7. doi: 10.1038/7445 10202937

[43] Rai KR Peterson BL Appelbaum FR Kolitz J Elias L Shepherd L. Fludarabine Compared With Chlorambucil as Primary Therapy for Chronic Lymphocytic Leukemia. N Engl J Med (2000) 343(24):1750–7. doi: 10.1056/NEJM200012143432402 11114313

[44] Ricci F Tedeschi A Morra E Montillo M. Fludarabine in the Treatment of Chronic Lymphocytic Leukemia: A Review. Ther Clin Risk Manag (2009) 5(1):187–207. doi: 10.2147/tcrm.s3688 19436622 PMC2697528

[45] Hansbro NG Horvat JC Wark PA Hansbro PM. Understanding the Mechanisms of Viral Induced Asthma: New Therapeutic Directions. Pharmacol Ther (2008) 117(3):313–53. doi: 10.1016/j.pharmthera.2007.11.002 PMC7112677 18234348

[46] DiVall MV Woolley AB. CHAPTER Pharmacologic Agents. Acute Care Handbook for Physical Therapists E-Book. St. Louis, Missouri: Elsevier Health Sciences (2019).

[47] Durbin JE Hackenmiller R Simon MC Levy DE. Targeted Disruption of the Mouse Stat1 Gene Results in Compromised Innate Immunity to Viral Disease. Cell (1996) 84(3):443–50. doi: 10.1016/S0092-8674(00)81289-1 8608598

[48] Yun NE Seregin AV Walker DH Popov VL Walker AG Smith JN. Mice Lacking Functional STAT1 Are Highly Susceptible to Lethal Infection With Lassa Virus. J Virol (2013) 87(19):10908–11. doi: 10.1128/JVI.01433-13 PMC3807383 23903830

[49] Zhang S Huo C Xiao J Fan T Zou S Qi P. P-STAT1 Regulates the Influenza A Virus Replication and Inflammatory Response in *Vitro* and *Vivo* . Virology (2019) 537:110–20. doi: 10.1016/j.virol.2019.08.023 31493649

[50] Gao C Wen C Li Z Lin S Gao S Ding H. Fludarabine Inhibits Infection of Zika Virus, SFTS Phlebovirus, and Enterovirus A71. Viruses (2021) 13(5):774. doi: 10.3390/v13050774 33925713 PMC8144994

