# Supplementary material for: Proteomic Analysis Reveals a Novel Therapeutic Strategy Using Fludarabine for Steroid-Resistant Asthma Exacerbation
Source: Front Immunol. 2022 Feb 25;13:805558. doi: 10.3389/fimmu.2022.805558 (PMC8913936; doi:10.3389/fimmu.2022.805558)
Supplement: Supplementary file 1 [file DataSheet_1.docx]

**Supplementary Figures**

**TITLE:** Proteomic analysis reveals a novel therapeutic strategy using Fludarabine for steroid-resistant asthma exacerbation

**Authors’ full names:** Xiaoming Liu^a,b+^, Xiang Li^a,b+^, Ling Chen^a,b^, Alan Hsu^b,c,d^, Kelly L. Asquith^a,b^, Chi Liu^e^, Karen Laurie^f^, Ian Barr^f^, Paul S. Foster^a,b,*+^, and Ming Yang^a,b,*+^

**Authors’ affiliation(s):**

^a^School of Biomedical Sciences and Pharmacy, College of Health, Medicine and Wellbeing, University of Newcastle, Callaghan, NSW, Australia.

^b^Priority of Research Center for Health Lungs, Hunter Medical Research Institute (HMRI), University of Newcastle, New Lambton Heights, NSW, Australia.

^c^School of Medicine and Public Health, College of Health, Medicine and Wellbeing, University of Newcastle, Callaghan, NSW, Australia.

^d^Programme in Emerging Infectious Diseases, Duke – National University of Singapore (NUS) Medical School, Singapore.

^e^Department of Physiology, School of Basic Medicine Science, Central South University, Changsha, Hunan, China.

^f^WHO Collaborating Centre for Reference and Research on Influenza, The Peter Doherty Institute for Infection and Immunity, Melbourne, VIC, Australia.

+Xiaoming Liu & Xiang Li contributed equally to this work and share first authorship; Ming Yang and Paul S. Foster contributed equally to this work and share last authorship.

**Short title:** Fludarabine inhibits asthma exacerbation

**+Correspondence:** Ming Yang and Paul S. Foster, Priority Research Centre for Healthy Lungs, Hunter Medical Research Institute, Lookout Road, New Lambton, NSW 2305, Australia. Emails: Ming.Yang@newcastle.edu.au, and Paul.Foster@newcastle.edu.au.

*
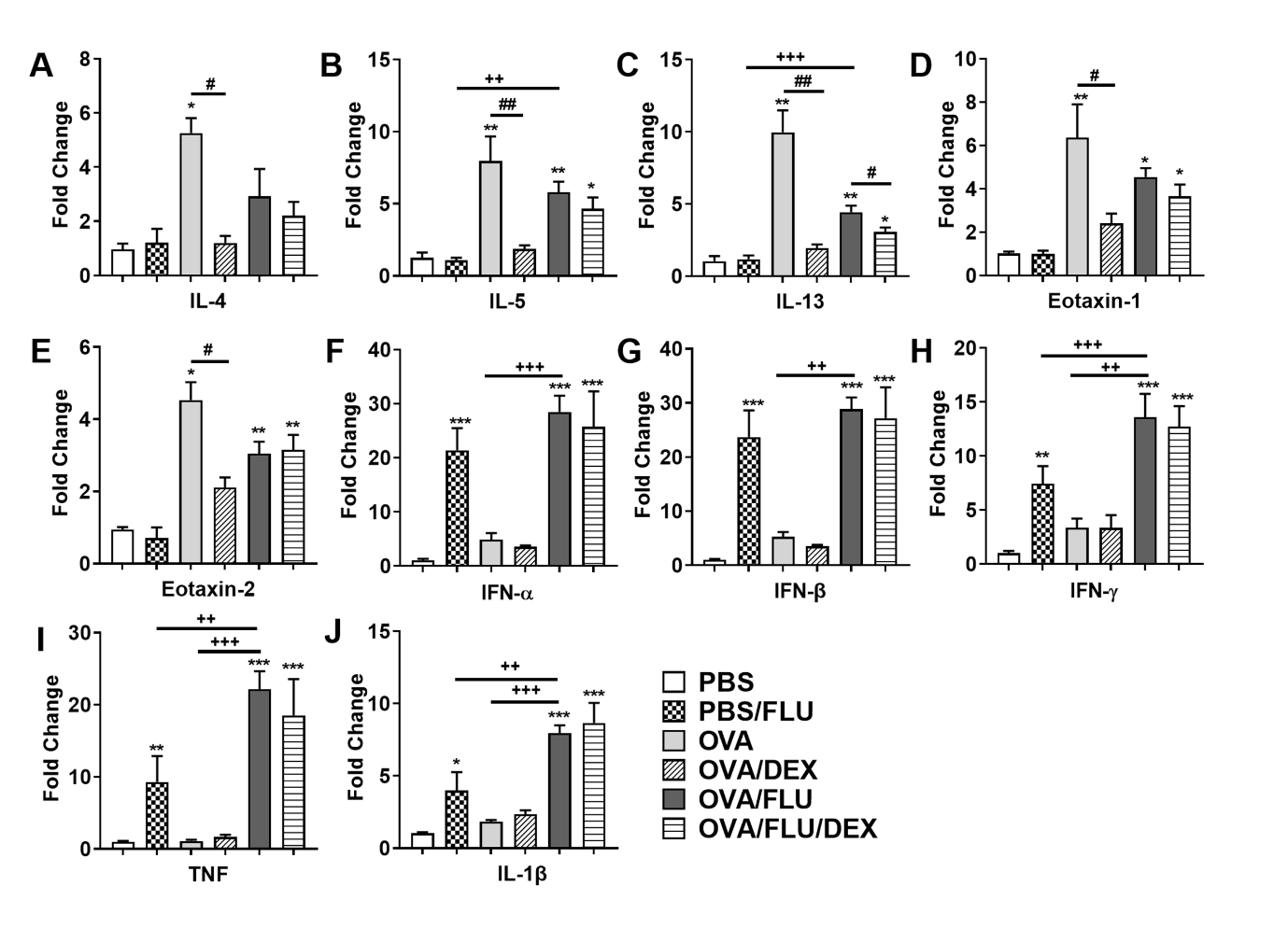
*

**Supplementary Figure 1.** **Effects of FLU infection and DEX on inflammatory cytokine expression in mice lungs with allergic asthma.** Cytokine production in lung tissues obtained from the same model as in Figure 1 was assessed on day 24 (5 days post infection). The mRNA levels of Th2 cytokines IL-4 (A), IL-5 (B), IL-13 (C), eotaxin-1 (D), eotaxin-2 (E); non-Th2 cytokines IFN-α (F), IFN-β (G), IFN-γ (H), TNF (I), IL-1β (J), were quantified by qPCR, the fold change was normalized to HPRT expression. Data are presented as mean ± SEM (n = 6-8 mice/group) and are representative of three independent experiments. *Designates significant differences from PBS-treated group (*p < 0.05, **p < 0.01, ***p < 0.001). #Designates significant differences from DEX-treated groups (#p < 0.05, ##p < 0.01). +Designates significant differences compared to OVA/FLU treated groups (++p < 0.01, +++p < 0.001).

**
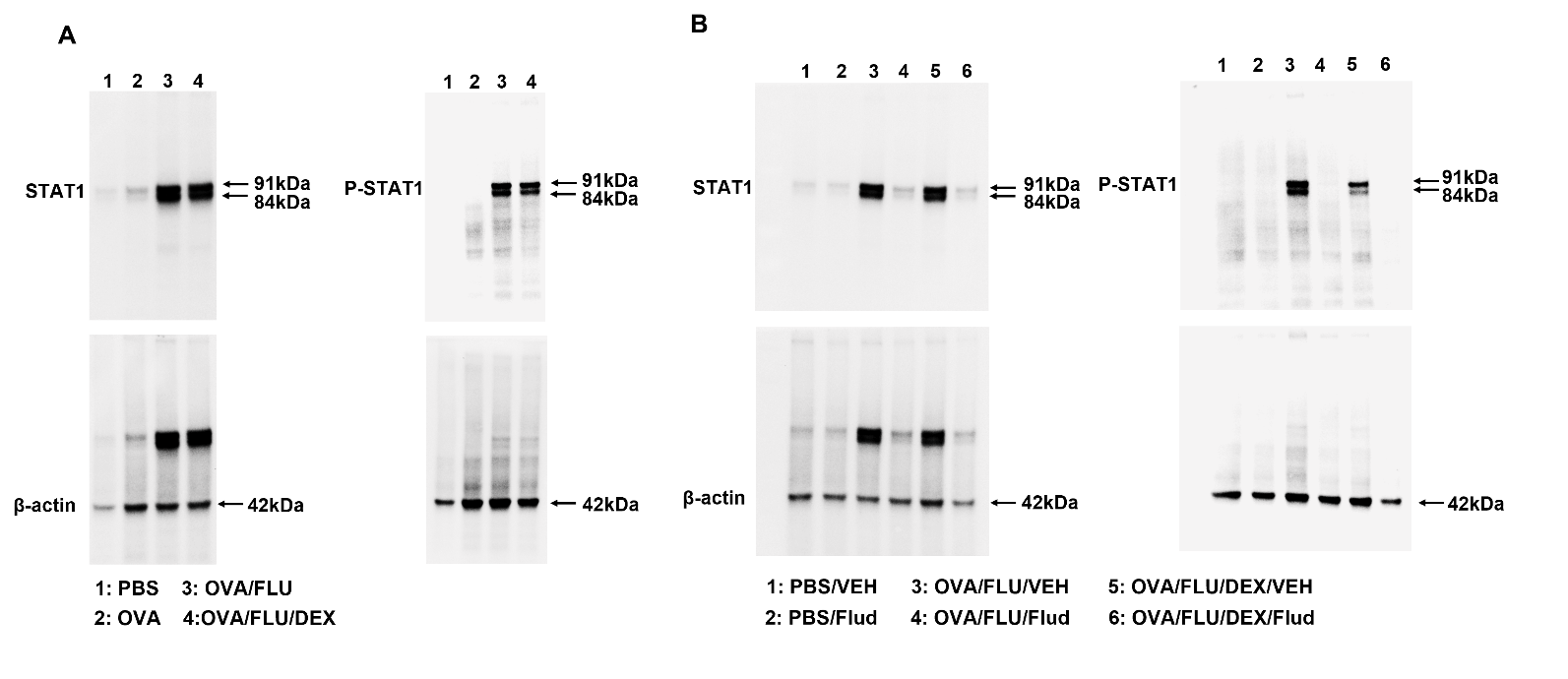
**

**Supplementary Figure 2.** **Whole blot presentation of STAT1 and phospho-STAT1 (P-STAT1) expression.** (A) 20 µg of lung proteins from each group as the same conditions in Figure 5 and (B) 20 µg of lung proteins from each group as the same conditions in Figure 6 were loaded on different stain-free gels and transferred to PVDF membranes after separation. Anti-STAT1 Ab and anti-phospho-STAT1 (Tyr701) Ab were firstly stained and visualized, then the blots were stripped to re-incubate with β-actin (42 kDa) as a loading control. Arrows indicated at 91 and 84 kDa were the two isoforms of STAT1 (Stat1α, Stat1β) and phospho-STAT1 (p-Stat1α and p-Stat1β).

*
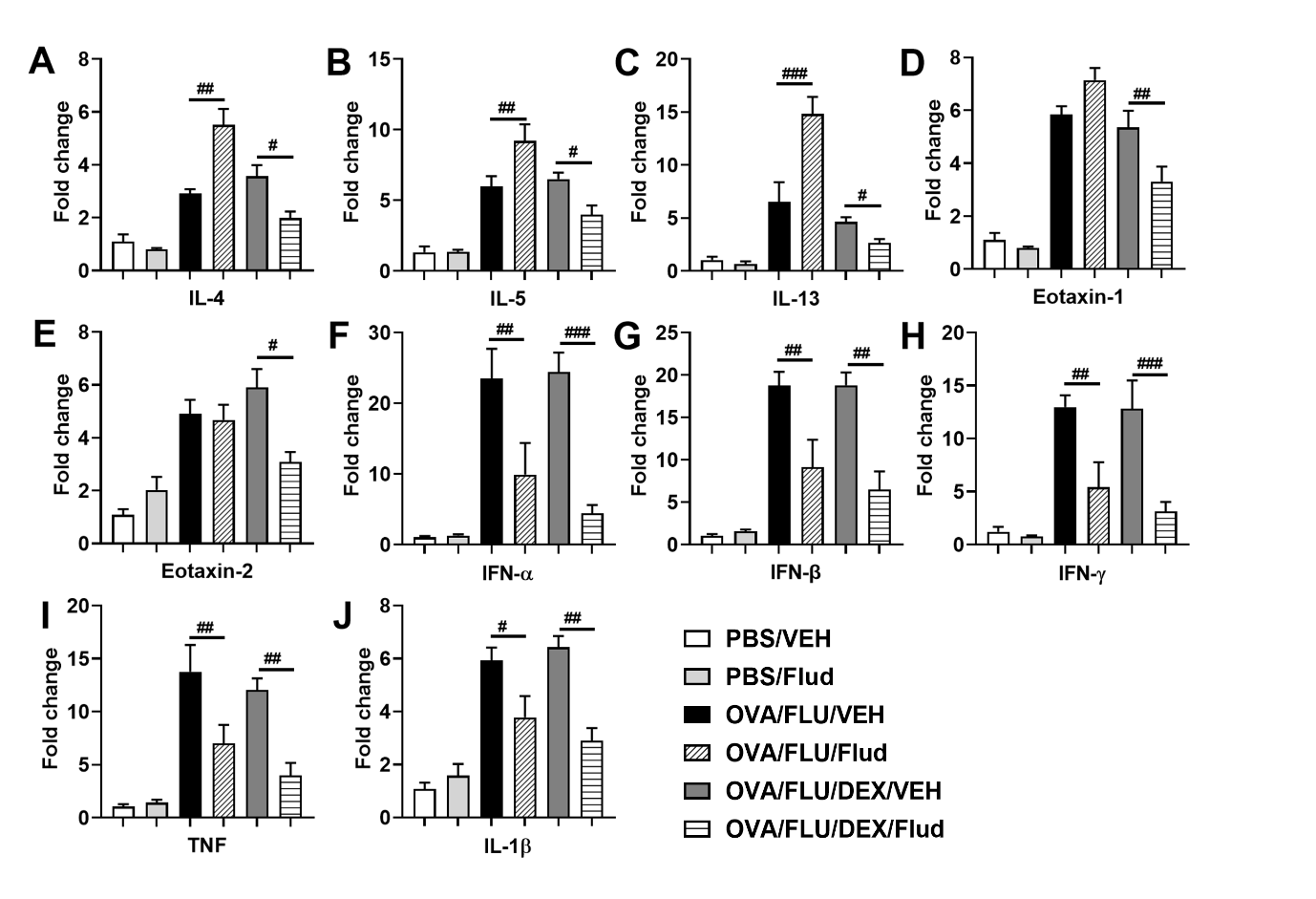
*

**Supplementary Figure 3. Effects of Fludarabine and its combination with DEX on inflammatory cytokines expression in mice lungs with FLU-induced asthma exacerbation.** Cytokine production in lung tissues obtained from same model in Figure 5 was assessed on day 24 (5 days post infection). The mRNA levels of Th2 cytokines IL-4 (A), IL-5 (B), IL-13 (C), eotaxin-1 (D), eotaxin-2 (E); non-Th2 cytokines IFN-α (F), IFN-β (G), IFN-γ (H), TNF (I), and IL-1β (J), were quantified by qPCR, the fold change was normalized to HPRT expression. Data are presented as mean ± SEM (n = 6-8 mice/group) and are representative of three independent experiments. Results are representative of three independent experiments. #Designates significant differences between Flud-treated and isotype-treated groups (#p < 0.05, ##p < 0.01, ###p < 0.001).
